# A developmental perspective on life with attention deficit hyperactivity disorder

**DOI:** 10.4102/sajpsychiatry.v24i0.1299

**Published:** 2018-10-17

**Authors:** Helen Clark

**Affiliations:** 1 Chris Hani Baragwanath Academic Hospital, South Africa

## Abstract

Attention deficit hyperactivity disorder (ADHD) is a lifelong neurodevelopmental disorder which impacts many aspects of function at each stage of the life journey through which it passes. Central to the understanding of this process is the recognition of the symptoms of ADHD itself and how they change over time. Beyond this the process itself has to be appreciated in terms of how this core integrates with parallel emerging patterns of comorbid disorders. The key to understanding how ADHD and its comorbidity integrate into and become part of the developing person is to realise that at each developing stage the symptoms of ADHD (as well as the comorbidity) will impact the overall development, creating backlogs or impairments which may ‘colour’ the persons as they grow and will not be as amenable to treatment as the ADHD or its comorbidity. If ADHD has this potential impact as the individual gets older, what are the treatment considerations in terms of early intervention?

